# Influence of Vitamin D on Islet Autoimmunity and Beta-Cell Function in Type 1 Diabetes

**DOI:** 10.3390/nu11092185

**Published:** 2019-09-11

**Authors:** Marco Infante, Camillo Ricordi, Janine Sanchez, Michael J. Clare-Salzler, Nathalia Padilla, Virginia Fuenmayor, Carmen Chavez, Ana Alvarez, David Baidal, Rodolfo Alejandro, Massimiliano Caprio, Andrea Fabbri

**Affiliations:** 1 Diabetes Research Institute (DRI) and Clinical Cell Transplant Program, University of Miami Miller School of Medicine, Miami, FL 33136, USA; cricordi@med.miami.edu (C.R.); nxp467@miami.edu (N.P.); vcf15@miami.edu (V.F.); cxc1759@miami.edu (C.C.); axa383@med.miami.edu (A.A.); dbaidal@med.miami.edu (D.B.); ralejand@med.miami.edu (R.A.); 2 Department of Systems Medicine, University of Rome “Tor Vergata”, Via Montpellier 1, 00133 Rome, Italy; andrea.fabbri@uniroma2.it; 3 Pediatric Endocrinology, University of Miami Miller School of Medicine, 1601 NW 12th Avenue, Miami, FL 33136, USA; jsanchez@med.miami.edu; 4 Department of Pathology, Immunology and Laboratory Medicine, University of Florida, 1600 SW Archer Rd, Gainesville, FL 32610, USA; salzler@ufl.edu; 5 Laboratory of Cardiovascular Endocrinology, IRCCS San Raffaele Pisana, Via di Val Cannuta 247, 00133 Rome, Italy; massimiliano.caprio@sanraffaele.it; 6 Department of Human Sciences and Promotion of the Quality of Life, San Raffaele Roma Open University, Via di Val Cannuta 247, 00166 Rome, Italy

**Keywords:** type 1 diabetes, T1D, autoimmunity, honeymoon phase, vitamin D, cholecalciferol, calcidiol, calcitriol, alfacalcidol, immunotherapy

## Abstract

Type 1 diabetes (T1D) is a chronic autoimmune disease leading to immune-mediated destruction of pancreatic beta cells, resulting in the need for insulin therapy. The incidence of T1D is increasing worldwide, thus prompting researchers to investigate novel immunomodulatory strategies to halt autoimmunity and modify disease progression. T1D is considered as a multifactorial disease, in which genetic predisposition and environmental factors interact to promote the triggering of autoimmune responses against beta cells. Over the last decades, it has become clear that vitamin D exerts anti-inflammatory and immunomodulatory effects, apart from its well-established role in the regulation of calcium homeostasis and bone metabolism. Importantly, the global incidence of vitamin D deficiency is also dramatically increasing and epidemiologic evidence suggests an involvement of vitamin D deficiency in T1D pathogenesis. Polymorphisms in genes critical for vitamin D metabolism have also been shown to modulate the risk of T1D. Moreover, several studies have investigated the role of vitamin D (in different doses and formulations) as a potential adjuvant immunomodulatory therapy in patients with new-onset and established T1D. This review aims to present the current knowledge on the immunomodulatory effects of vitamin D and summarize the clinical interventional studies investigating its use for prevention or treatment of T1D.

## 1. Introduction: Pathogenesis and Natural History of Type 1 Diabetes

Type 1 diabetes (T1D) is an organ-specific chronic autoimmune disease leading to immune-mediated destruction of insulin-secreting beta cells within the pancreatic islets, resulting in lifelong dependence on exogenous insulin [1,2]. Insulitis is the inflammatory lesion considered as the histological hallmark of T1D; it consists of the infiltration of pancreatic islets by macrophages, T helper cells (CD4+ or Th cells), and cytotoxic T cells (CD8+), ultimately resulting in the destruction of beta cells [3]. Notably, CD4+ T cells mediate the triggering of the autoimmune process and promote recruitment and activation of CD8+ T cells within the pancreatic islets. In turn, autoreactive CD8+ T cells recognize major histocompatibility complex (MHC) class I-restricted islet autoantigens on the beta-cell surface and exert their cytotoxic effects through several effector mediators, including Th1 cytokines (e.g., TNF-α, IFN-γ) [4,5]. Although T1D has been traditionally considered as a Th1-mediated pathology, growing evidence suggests a relevant role of Th17 cells [5,6]. Moreover, defects in the ability of regulatory T cells (Tregs) to suppress activity and proliferation of CD4+ and CD8+ T cells have also been reported [7,8,9].

According to the new staging classification system [10,11,12], T1D can be categorized into four sequential stages:

- Stage 1: Subjects exhibit islet autoimmunity, as evidenced by the persistent presence of at least two islet autoantibodies [autoantibodies directed against insulin, glutamic acid decarboxylase (GAD65), insulinoma-associated antigen 2 (IA-2), or zinc transporter-8 (ZnT8)], but remain normoglycemic and asymptomatic.
- Stage 2: Subjects maintain multiple islet autoantibody positivity and remain asymptomatic, but exhibit dysglycemia, as evidenced by impaired fasting glucose, an abnormal oral glucose tolerance test (OGTT), or HbA1c (glycated hemoglobin) ≥5.7% [13].
- Stage 3: Subjects experience the onset of clinical T1D, which is often accompanied by symptoms (polyuria, polydipsia, fatigue, weight loss, diabetic ketoacidosis, etc.) [10,12], and occurs upon the loss of approximately 70–80% of beta-cell mass [14].
- Stage 4: Established/long-term disease [11].

The clinical onset of T1D usually occurs several years after the initiation of the beta-cell destruction process [15] and becomes evident predominantly in childhood and young adulthood, although it can be observed at any age [16,17]. A few weeks after clinical onset of the disease and insulin therapy initiation, most newly-diagnosed T1D patients experience a transient and partial spontaneous remission phase, also known as the “honeymoon phase” [18]. During this phase, the remaining beta cells are still able to produce enough insulin leading to a relevant reduction in exogenous insulin requirements and near-normal glycemic control. In a few cases, complete remission can occur, resulting in insulin independence. Complete and partial remissions have been described in approximately 2–12% and 18–62% of young T1D patients, respectively [19]. The duration of remission ranges broadly between one month and 13 years [20], with an average of seven months [21]. Older age (>five years) and less severe clinical manifestations at diagnosis have been related to highest rates and duration of remission phase [18,21,22]. The pathogenesis of the remission phase is still not completely understood, even though the potential involvement of a transient development of adaptative immune tolerance and/or beta-cell regeneration and recovery due to a more favorable immunological environment has been suggested, along with an improvement in glycemic control, glucotoxicity, and insulin sensitivity obtained through insulin therapy [18,23]. Hence, this phase holds a remarkable clinical significance, since it can be targeted to investigate the potential efficacy of different therapeutic agents in halting or slowing down the autoimmune process and the disease progression in T1D [18]. 

T1D is considered as a complex multifactorial disease, in which environmental factors and genetic predisposition interact to promote the triggering of an autoimmune response against beta cells [24]. Even though the specific events that initiate T1D are still not completely understood, several genetic and environmental factors have been described as risk factors for development of the disease [25,26,27]. Among genetic factors, human leukocyte antigen (HLA) class II haplotypes DR3-DQ2 and DR4-DQ8 confer the highest risk of T1D [26]. However, environmental factors appear to be necessary for triggering islet autoimmunity and promoting T1D onset, especially in individuals with genetic susceptibility to the disease [27]. Main candidate triggers include some viruses (especially enteroviruses) [28,29,30], gut microbiota [31], dietary factors [32], and toxins (e.g., nitrites, nitrates, nitrosamines, etc.) [33,34,35,36]. 

Importantly, incidence of T1D among younger children has substantially increased over the last decades and it is expected to rise during the next years [37,38]. This increasing trend could be explained, at least in part, by recent modifications in environment and/or lifestyle habits [27]. Of note, the parallel growing increase in worldwide incidence of vitamin D deficiency across all age groups—including children and adolescents [39,40]—poses some questions about the putative involvement of vitamin D deficiency in T1D pathophysiology. This review aims to describe the immunomodulatory effects of vitamin D and to provide an overview of the studies evaluating the impact of vitamin D status, vitamin D genetic polymorphisms and vitamin D supplementation in modulating the risk of T1D. Moreover, we will summarize previous and ongoing clinical interventional studies on the use of vitamin D as an adjuvant immunomodulatory therapy in patients with new-onset and established T1D. 

## 2. Chemical and Pharmacokinetic Properties of Vitamin D

**Figure 1:**
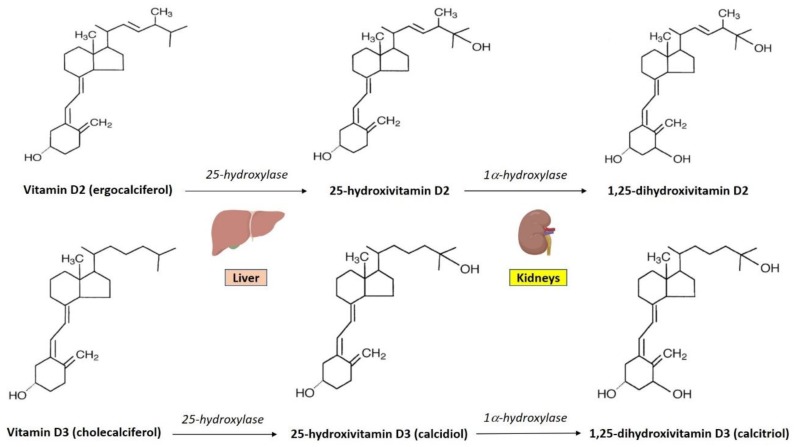
Chemical structure of the main forms of vitamin D and synthesis of biologically-active metabolites (1,25-dihydroxivitamin D2 and 1,25-dihydroxivitamin D3).

Vitamin D is a term that generally refers to a group of fat-soluble secosteroids (open-ring steroids). Ergocalciferol (vitamin D2) is produced by ultraviolet irradiation of the plant sterol ergosterol, whereas cholecalciferol (vitamin D3) is synthesized in the human skin upon ultraviolet-B (UVB) light irradiation of its precursor 7-dehydrocholesterol (7-DHC). In terms of chemical structure, vitamin D2 differs from vitamin D3 in having a methyl group at C24 and a double bond between C22 and C23 in the isoprenoid side chain [41] (Figure 1). 

Vitamin D2 and vitamin D3 have a short serum half-life (approximately 24 h) [42] and are lipophilic compounds that partly accumulate in the adipose tissue [43,44]. After oral ingestion, vitamin D2 and vitamin D3 are gradually converted into their biologically-active forms through two sequential hydroxylations at C25 and C1, which result in the synthesis of 25-hydroxivitamin D2, 25-hydroxivitamin D3 (25(OH)D3, referred to as calcidiol), 1,25-dihydroxivitamin D2, and 1,25-dihydroxivitamin D3 (1,25(OH)2D3, referred to as calcitriol) [41] (Figure 1). In humans, 25(OH)D3 is the major circulating form of vitamin D, and total 25(OH)D serum concentrations represent the most reliable biomarker of vitamin D status due to the longer serum half-life of 25(OH)D3 (~15 days) compared to vitamin D3 (~24 h) and calcitriol (~15 h) [45,46].

## 3. Vitamin D Synthesis and Metabolism

In humans, vitamin D is predominantly produced in the skin during exposure to the sunlight. However, a small proportion of vitamin D (~20%) is obtained through diet [47], which provides the two major forms of vitamin D, namely vitamin D2, contained in yeast and fungi, and vitamin D3, contained in a few animal sources, including fatty fish and cod liver oil [39,48]. Sunlight exposure only provides vitamin D in the form of vitamin D3, which is produced in skin from 7-DHC upon sunlight exposure. Vitamin D3 is then transported to the liver by vitamin D-binding protein (VDBP), an alpha-globulin mainly synthesized and secreted by the liver, which acts as the transporter of vitamin D metabolites in the circulation. In the liver, vitamin D-25-hydroxylase enzyme catalyzes the conversion of vitamin D3 into 25-hydroxyvitamin D3. Then, 25(OH)D3 is transported to the kidneys, where 1α-hydroxylase enzyme converts it into 1,25-dihydroxyvitamin D3, which is the biologically-active metabolite of vitamin D [49] (Figure 1). In order to control calcitriol circulating levels, the mitochondrial enzyme 24-hydroxylase catalyzes the hydroxylation of 25(OH)D3 or 1,25(OH)2D3 on carbon 24 (C24), leading to the synthesis of the less active metabolites 24,25(OH)2D3 and 1,24,25(OH)3D3, respectively [50]. Finally, calcitriol initiates its signaling cascade by binding to the nuclear vitamin D receptor (VDR), which forms a heterodimer with retinoid X receptor (RXR) and binds to specific DNA sequences (also known as VDREs, vitamin D response elements), regulating the transcription of several genes [49]. Notably, VDR has been detected in almost all human cells (including immune cells) [51] and vitamin D has been shown to exert several pleiotropic effects beyond the well-known regulation of calcium homeostasis and bone metabolism [52,53,54].

## 4. Immunomodulatory Effects of Vitamin D

Functional VDR has been identified in almost all immune cells, including antigen-presenting cells (APCs) and T lymphocytes [55,56], thus providing an indirect evidence of vitamin D action on immune system. Moreover, immune cells—especially APCs (activated macrophages and dendritic cells)—express the enzyme 1α-hydroxylase [57,58] and are thereby able to synthesize and secrete calcitriol under specific immune stimuli, such as interferon-gamma (IFN-γ) [59].

**Figure 2:**
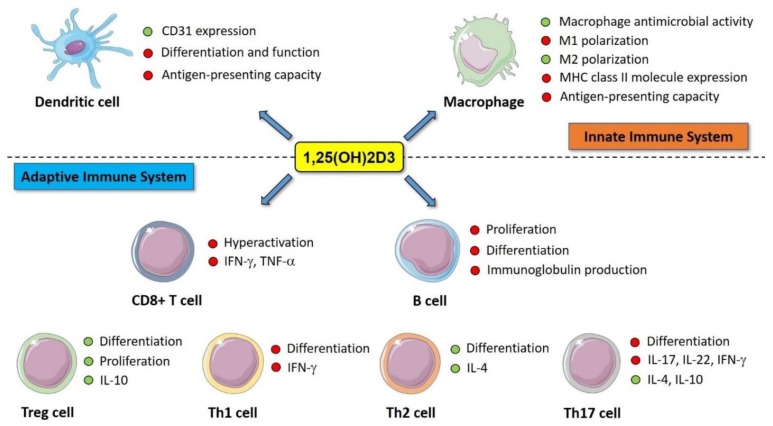
An overview of the anti-inflammatory and immunomodulatory effects exerted by the active metabolite of vitamin D 1,25(OH)2D3 (referred to as calcitriol) on innate and adaptive immune system. Red dots represent downregulation, whereas green dots represent upregulation. Abbreviations: IFN-γ, interferon gamma; IL-4, interleukin 4; IL-10, interleukin 10; IL-17, interleukin 17; IL-22, interleukin 22; M1, classically-activated macrophages; M2, alternatively-activated macrophages; MHC, major histocompatibility complex; TNF-α, tumor necrosis factor-alpha.

Vitamin D exerts its action on both innate and adaptive immune system through VDR [52,55,60]. Overall, the immunomodulatory effects of vitamin D mostly depend upon the capacity of its biologically-active form calcitriol to regulate expression of several genes involved in cell proliferation, differentiation, and function [52,61,62]. Calcitriol downregulates the adaptive immune responses, promoting the induction of immunological tolerance and exerting anti-inflammatory effects through different mechanisms. Of note, calcitriol inhibits differentiation, maturation, and function of dendritic cells (DCs), rendering them more tolerogenic and unable to act as mature APCs [63,64,65,66,67]. It has recently been demonstrated that calcitriol can modulate DC function upregulating the expression of CD31 (a member of the immunoglobulin superfamily), leading to a decreased in vitro ability to prime CD4+ T cells by preventing a stable cell–cell contact [68]. On the other hand, calcitriol stimulates differentiation and activation of macrophages and promotes their antimicrobial activity, enhancing chemotaxis and phagocytosis and stimulating the local production of defensins (e.g., cathelicidin and β2-defensin) [69,70]. However, calcitriol reduces macrophage antigen-presentation and T-cell stimulatory capacity by reducing the surface expression of MHC class II molecules [52,57,71]. In addition, calcitriol promotes the shift of macrophage polarization from a pro-inflammatory phenotype (M1 or “classically activated” macrophages) towards an anti-inflammatory one (M2 or “alternatively activated” macrophages) [72], and inhibits the expression of pro-inflammatory cytokines by monocytes and macrophages [73]. These changes ultimately result in the inability of APCs to present antigens, thus leading to T cell anergy and impaired B cell proliferation, differentiation into plasma cells and immunoglobulin production [74]. However, a direct inhibitory effect of calcitriol on B cell differentiation and immunoglobulin production has also been reported [75,76]. Calcitriol up-regulates Tregs [77] and affects Th cell polarization by increasing Th2 cells and inhibiting Th1 and Th17 cell development, thus stimulating a shift of T cells from an “effector” towards a “regulatory” phenotype [52,56,78,79,80]. Moreover, calcitriol acts on CD8+ T cells, preventing their hyperactivation and secretion of IFN-γ and tumor necrosis factor-alpha (TNF-α) [81,82]. In light of all the aforementioned mechanisms, calcitriol regulates cytokine production by immune cells, increasing production of anti-inflammatory cytokines (e.g., IL-4, IL-10) and decreasing pro-inflammatory cytokines (e.g., IL-1β, IL-2, IL-6, IL-17, IL-22, TNF-α, IFN-γ) [56,82,83,84,85,86,87]. Figure 2 illustrates the anti-inflammatory and immunomodulatory effects exerted by calcitriol. 

## 5. Role of Vitamin D in Islet Autoimmunity, Inflammation, and Beta-Cell Function: Evidence from Pre-Clinical Studies

Several pre-clinical studies showed an involvement of vitamin D deficiency in islet autoimmunity and disease progression in autoimmune diabetes. The majority of these studies have been conducted in non-obese diabetic (NOD) mice, which have long been used as an animal model of human T1D [88]. It has been demonstrated that vitamin D deficiency in early life leads to higher incidence and earlier onset of diabetes in NOD mice [89]. NOD mice also show defects in macrophage 1α-hydroxylase up-regulation mediated by immune stimuli, such as lipopolysaccharide (LPS) and IFN-γ [57]. Importantly, calcitriol and its analogues have been shown to prevent diabetes and insulitis in NOD mice particularly when administered at an early age (when insulitis and beta-cell immune-mediated attack have not yet occurred) [90,91,92], although they are still able to arrest diabetes progression when administered at an older age and in presence of a more advanced disease phase [93,94]. Mathieu et al. [90,92] documented that long-term treatment with high doses of calcitriol (5 μg/kg given daily or on alternate days) is able to reduce the incidence of insulitis and diabetes in NOD mice without major side effects. Another study conducted on NOD mice showed that short-term administration of a calcitriol analogue inhibits LPS-induced interleukin (IL)-12 and IFN-γ production, arrests Th1 cell infiltration within the pancreatic islets, enhances the frequency of CD4+CD25+ regulatory T cells within the pancreatic lymph nodes, curbs the progression of insulitis, and prevents development of diabetes at non-hypercalcemic doses [94]. A remarkable shift in the cytokine secretion profile from predominantly Th1 (IFN-γ) to Th2 (IL-4) in NOD mice treated with calcitriol has also been shown [78]. Further studies conducted on NOD mice found that calcitriol restores the sensitivity of T lymphocytes to apoptosis-inducing signals, thus promoting the elimination of autoimmune effector cells [95,96]. In addition, DCs exposed to calcitriol or its analogue TX527 have been shown to alter the response pattern of GAD65-specific autoreactive T cell clones, inhibiting cell proliferation and promoting apoptosis [97,98]. Similar results have also been described with the use of vitamin D3, which can be locally converted into the active form by immune cells themselves and is therefore associated with a lower risk of toxicity and calcemic side effects than calcitriol. Takiishi et al. [99] showed that NOD mice fed an enriched-vitamin D3 diet (800 IU/day) lifelong (from three weeks until 35 weeks of age) exhibited a significant reduction in diabetes development, which correlated with higher pancreatic insulin content and less severe insulitis compared to the control group. The authors also showed that vitamin D3 supplementation led to a phenotypic shift from effector T cells (IFN-γ-expressing CD4+ and CD8+ T cells) to Tregs in pancreatic lymph nodes and islet infiltrates. 

Inflammation plays an important role in T1D pathogenesis, contributing to beta-cell dysfunction and apoptosis through cytokines and chemokines produced by both beta cells and immune cells [100]. In this regard, calcitriol and its analogues have been shown to prevent the IL-1β-induced inhibition of beta-cell function, as well as IFN-γ-stimulated beta-cell expression of MHC class I and class II molecules in isolated rat pancreatic islets [101,102]. In addition, it has been shown that calcitriol increases the levels of the antiapoptotic protein A20 and reduces IL-6 production, nitric oxide synthesis, and MHC class I expression in isolated human pancreatic islets exposed to pro-inflammatory cytokines, such as IL-1β, TNF-α, and IFN-γ [103,104]. Wei et al. [105] recently documented that association of VDR with the alternative chromatin remodeling complex PBAF enhances the VDR-dependent transcriptional program, resulting in reduced cytokine-induced beta-cell pro-inflammatory response and preserved beta-cell function in human beta-like cells and in diabetic mouse models.

Compelling evidence also suggests a role of vitamin D in beta-cell function and insulin secretion [106]. Norman et al. [107] first demonstrated that vitamin D deficiency inhibits insulin secretion in perfused pancreases isolated from rats. Moreover, human pancreatic beta cells express both 1α-hydroxylase [108] and VDR [109], and a VDRE has been detected in the human insulin gene promoter [110]. Zeitz et al. [111] showed that mice lacking a functional VDR exhibit an impaired insulin secretory capacity compared to wild-type controls. Importantly, the animals were fed a rescue calcium-enriched diet to exclude possible influences of hypocalcemia on pancreatic endocrine function. Accordingly, Bourlon et al. [112] demonstrated that calcitriol promotes de novo insulin biosynthesis and accelerates the conversion of proinsulin to insulin in rat pancreatic islets. Furthermore, vitamin D supplementation has been shown to reverse the defects in insulin secretion observed in mice and rabbits with vitamin D deficiency [107,113,114,115]. 

Altogether, these pre-clinical findings suggest that vitamin D and its analogues can preserve beta-cell mass and function from immune-mediated attack through different mechanisms, such as: (i) promoting the shift from a Th1 to a Th2 cytokine expression profile, (ii) enhancing the clearance of autoreactive T cells and decreasing the Th1 cell infiltration within the pancreatic islets, (iii) reducing the cytokine-induced beta-cell damage, as well as the beta-cell expression of MHC class I and class II molecules, and (iv) promoting Tregs differentiation and suppressor capacity.

## 6. The Role of Polymorphisms of Vitamin D Metabolism Genes in T1D

Aside from sunlight exposure, dietary habits, and vitamin D supplementation, different polymorphisms of genes involved in vitamin D metabolism—especially those encoding vitamin D hydroxylases, VDBP and VDR—may influence the risk of islet autoimmunity and T1D. 

Ramos-Lopez et al. [116] documented an association of single nucleotide polymorphisms (SNPs) in *CYP2R1*—the gene encoding the vitamin D 25-hydroxylase—in patients with T1D and with their serum 25(OH)D levels, suggesting that the G allele of the rs10741657 SNP is associated with T1D susceptibility, whereas the A allele of the same SNP might confer protection against disease development. Accordingly, Cooper et al. [117] showed a significant association of both rs10741657 and rs12794714 SNPs in *CYP2R1* and T1D risk. A large case-control study conducted by Bailey et al. [118] on 7,854 patients with T1D and 8,758 healthy controls from Great Britain, provided evidence for the association of two SNPs (rs10877012 and rs4646536) in *CYP27B1*—the gene encoding the vitamin D 1α-hydroxylase—with T1D. Furthermore, the C allele of rs10877012 SNP in *CYP27B1* was significantly associated with an increased risk of T1D [118]. In keeping with these findings, Hussein et al. [119] reported that GG genotype of *CYP2R1* (SNP rs10741657) or CC genotype of *CYP27B1* (SNP rs10877012) increased the risk of developing T1D in Egyptian children. Interestingly, subjects carrying both genotypes showed a significantly higher risk of T1D compared to those carrying only one of them, thus indicating a potential synergism between GG genotype of *CYP2R1* and CC genotype of *CYP27B1* in determining the risk of T1D. Moreover, serum 25(OH)D levels were significantly lower in subjects carrying *CYP2R1* GG genotype and *CYP27B1* CC genotype compared to those carrying *CYP2R1* AA genotype and *CYP27B1* AA genotype, respectively [119]. However, other studies did not confirm these results. For instance, Thorsen et al. [120] did not find an association between SNPs in *CYP2R1* and *CYP27B1* (rs10741657 and rs4646536, respectively) and risk of T1D in a juvenile Danish population. Furthermore, an association between rs6013897 SNP in *CYP24A1*—the gene encoding the vitamin D-inactivating enzyme 24-hydroxylase—and risk of T1D has not been found [117,118,120].

Some studies reported a relationship between genetic polymorphisms of VDBP and T1D [121,122] (as a likely consequence of impaired VDBP affinity for its ligand 1,25-dihydroxyvitamin D and subsequent alteration in 1,25-dihydroxyvitamin D concentrations) [123], even though other studies did not confirm these findings [120,124]. Nevertheless, evidence indicates that VDBP may represent an additional player in T1D pathogenesis. A retrospective cross-sectional study demonstrated that T1D patients exhibited lower serum VDBP levels compared to healthy controls, although VDBP levels did not associate with serum 25(OH)D levels, age, or disease duration [124]. Higher maternal plasma VDBP levels post-partum have also been associated with significantly lower risk of T1D in offspring [125]. Intriguingly, an expression-based genome-wide association study, aimed at identifying additional antigenic proteins involved in T1D pathogenesis, suggested VDBP as a potential candidate [126]. In fact, T-cell proliferation assays showed stronger T-cell reactivity against VDBP compared to control stimulations in NOD mice. Moreover, T1D patients showed higher levels and frequencies of serum anti-VDBP autoantibodies (VDBP-Abs) compared to healthy controls, and VDBP-Ab levels were inversely correlated with serum 25(OH)D levels in patients who developed T1D during winter. Immunohistochemical analysis revealed that VDBP was highly and specifically expressed in alfa cells of pancreatic islets from pancreatic tissue sections of human subjects with islet autoantibody positivity and prediabetic NOD mice [126].

A potential role of polymorphisms of VDR gene in T1D has also been suggested. Norris et al. [127] showed that higher serum 25(OH)D levels are associated with lower risk of islet autoimmunity in children at increased genetic risk of T1D. Interestingly, the association between childhood 25(OH)D status and islet autoimmunity was modified by the rs7975232 SNP in *VDR*, where for each additional minor allele higher 25(OH)D concentrations were associated with a greater reduction in islet autoimmunity risk. This indicates that vitamin D and VDR play a combined role in the development of islet autoimmunity among children with increased genetic risk for T1D [127]. In addition, higher cord blood 25(OH)D levels at birth have recently been shown to predict a lower risk of developing T1D in children homozygous for the VDR rs11568820 G/G genotype [125]. Several studies have demonstrated an association between increased T1D risk and certain SNPs in *VDR* (especially Bsm-I and Fok-I), although the exact alleles that most predispose to T1D development remain still controversial [128,129,130,131,132,133,134]. Finally, Habibian et al. [134] showed that sufficient serum 25(OH)D levels (≥30 ng/mL) and certain genotypes of TaqI and BsmI SNPs in *VDR* were significantly associated with higher levels of stimulated C-peptide in patients with new-onset T1D, potentially resulting in a greater preservation of residual beta-cell mass and function. 

Overall, these findings suggest that SNPs in genes critical for synthesis, transport, and action of vitamin D may affect the risk of T1D development. In particular, these polymorphisms may be associated with decreased VDR, 25-hydroxylase, and 1α-hydroxylase activity and expression, along with reduced affinity of VDBP for vitamin D metabolites, potentially affecting the circulating levels of vitamin D and its immunomodulatory effects. Future prospective studies are therefore needed in order to better investigate the relationship between T1D pathogenesis and SNPs in genes involved in vitamin D metabolism, as well as to identify polymorphisms that may require different doses of vitamin D to achieve target serum levels required for vitamin D-related immunomodulatory effects. Moreover, the interaction of these polymorphisms among each other and with various environmental factors will also need to be taken into account. 

## 7. Role of Vitamin D Status and Vitamin D Supplementation in T1D: Epidemiologic Evidence

Apart from the aforementioned pre-clinical evidence for the protective effects of vitamin D against beta-cell dysfunction, islet autoimmunity, and inflammatory responses, epidemiologic data suggest a potential association between hypovitaminosis D and T1D. An increase in worldwide prevalence and incidence of vitamin D deficiency and T1D has been observed over the last years [37,39,40,135,136,137].

The DIAMOND Project Group found a higher incidence of T1D (data collected from 1990 to 1994) in certain regions at a higher latitude (with low UVB irradiance), such as Finland (36.5/100,000 per year), Sweden (27.5/100,000 per year), and Norway (21.2/100,000 per year) [138]. Some studies documented a seasonal pattern of T1D onset, consisting of cyclic incidence peaks during winter, early spring, and late autumn, associated with summer pauses [139,140,141]. Moreover, Mohr et al. [142] found that low UVB irradiance was associated with significantly higher incidence rates of T1D in childhood. The same authors showed a gradual rise in incidence rates of T1D in Finland (from 18/100,000 population in 1965 to 64/100,000 in 2005), which paralleled the progressive reduction in official Finnish daily vitamin D intake recommendations during the same period [143]. 

Individuals with new-onset and established T1D exhibited significantly lower levels of 25(OH)D compared to healthy controls in several observational studies [144,145,146,147,148,149,150,151,152,153]. As previously mentioned, Norris et al. [127] have recently shown that higher serum 25(OH)D levels are associated with lower risk of islet autoimmunity in children at increased genetic risk for T1D. Furthermore, Raab et al. [154] documented that prediabetic children with multiple islet autoantibodies have reduced 25(OH)D levels compared to autoantibody-negative subjects, although they did not show a faster progression to T1D over a of 5.8-year median observation period. Intriguingly, Federico et al. [151] reported that newly-diagnosed enterovirus-positive T1D children and adolescents had a more prevalent and profound vitamin D deficiency compared to their virus-negative counterparts, suggesting that an inadequate vitamin D status could be associated with a higher risk for enterovirus infection. Importantly, enteroviruses have been suggested as potential environmental (infectious) factors triggering the development of T1D in presence of genetic susceptibility to the disease [28]. 

Cadario et al. [155] found no association between serum 25(OH)D levels at birth in newborns and risk of developing T1D up to 10 years of age. Similar results were observed by Mäkinen et al. [156], who did not find significant differences in serum 25(OH)D levels (median age at blood sampling: three years) between children who progressed to T1D and children who did not develop the disease. In contrast with these findings, vitamin D status appears to exert a greater impact on T1D risk among young adults. Gorham et al. [157] showed that US military service members with lower serum 25(OH)D levels had a higher risk to develop insulin-requiring diabetes compared to those with higher concentrations. The risk of insulin-requiring diabetes was 3.5-fold higher in individuals with serum 25(OH)D levels within the lowest quintile (<17.2 ng/mL) compared to those with serum 25(OH)D levels within the highest quintile (≥40 ng/mL). Moreover, the mean serum 25(OH)D levels were significantly lower in individuals who developed diabetes compared to healthy controls (24.8 ng/mL vs. 29 ng/mL, respectively; *p* < 0.0001). The median interval between blood sample collection and diabetes diagnosis was one year. Nevertheless, the main limitation of this study consists in the lack of assessment of islet autoantibodies, thus preventing the distinction between T1D and type 2 diabetes (T2D). Another nested case-control study conducted by Munger et al. [158] on American active-duty military personnel reported that non-Hispanic whites with average serum 25(OH)D levels ≥40 ng/mL had a significant 44% lower risk of developing T1D compared to those with average serum 25(OH)D levels <30 ng/mL during an average follow-up of 5.4 years. Nonetheless, no significant association between vitamin D status and T1D risk was observed among non-Hispanic blacks or Hispanics [158].

Although the cause and effect relationship between vitamin D deficiency and T1D has yet to be widely investigated, the role of vitamin D in determining the risk of T1D seems to be variable across the different stages of life. In this regard, several studies have evaluated the impact of vitamin D intake (via food sources or supplements) during pregnancy and/or early life on the prevalence of T1D. Notably, studies evaluating the relationship between vitamin D status and/or vitamin D intake during pregnancy and the risk of T1D in the offspring are still inconclusive. Some studies showed that maternal intake of vitamin D-enriched food (e.g., cod liver oil) during pregnancy is associated with a reduced risk of islet autoimmunity [159] and T1D [160] in the offspring. A case-control study nested within a cohort of 29,072 women in Norway showed that the mothers of children who developed T1D before 15 years of age had significantly lower serum 25(OH)D levels (measured from serum samples mainly collected during the last trimester of pregnancy) compared to the mothers of children who did not develop the disease [161]. Moreover, the odds ratio of T1D was more than two-fold higher in children born from women with serum 25(OH)D levels in the first quartile (≤21.6 ng/mL) compared to children born from women with serum 25(OH)D levels within the fourth quartile (>35.6 ng/mL) [161]. On the contrary, a case-control study conducted on the Finnish Maternity Cohort found no difference in serum 25(OH)D levels during the first trimester of pregnancy between mothers of children who subsequently developed T1D and mothers of non-diabetic children of the same age (mean age of children at T1D diagnosis was 3.4 years; range 0–7 years) [162]. Marjamäki et al. [163] reported that maternal intake of vitamin D from food or supplements during pregnancy was not associated with islet autoimmunity or risk of T1D in a population-based Finnish birth cohort of infants at genetic risk of T1D. In the ABIS (All Babies in Southeast Sweden) study, vitamin D supplementation during pregnancy was associated with reduced risk of islet autoimmunity in the offspring at one year of age, but not at 2.5 years of age [164]. The use of vitamin D supplements during pregnancy was not related to the risk of developing T1D in children before 14–16 years of age in the same cohort [165]. A meta-analysis of observational studies showed no significant association between maternal intake of cod liver oil or vitamin D and risk of T1D in the offspring [166]. Accordingly, Silvis et al. [167] recently showed that vitamin D supplementation during pregnancy was not associated with risk for islet autoimmunity in the offspring among the TEDDY (The Environmental Determinants of Diabetes in the Young Study) prospective cohort of children with increased genetic risk for T1D. 

On the other hand, various studies indicate that vitamin D intake during early childhood may have an impact in reducing the risk of T1D later in life. A large birth-cohort Finnish study first demonstrated that dietary vitamin D supplementation during the first year of life was associated with a reduced frequency of T1D [168]. Moreover, regular vitamin D supplementation at a dose of 2000 IU/day was associated with a significant 78% reduction in risk of developing T1D compared to vitamin D intake at a lower dose (<2000 IU/day) [168]. A large multicenter, population-based case-control study on early risk factors of childhood onset T1D conducted by the EURODIAB Substudy 2 Study Group showed that vitamin D supplementation in early infancy (data collected by standardized questionnaires or interviews) is associated with a reduced risk of developing T1D in later life [169]. Similar findings were subsequently confirmed by two meta-analyses of observational studies on vitamin D intake and risk of T1D, which found that vitamin D supplementation in infancy was associated with an approximately 20% reduction in the risk of T1D [166,170]. Furthermore, a Norwegian case-control study conducted by Stene et al. [171] highlighted the importance of the timing of vitamin D intake. Indeed, infants supplemented with cod liver oil between seven and 12 months of age exhibited a significant lower risk of T1D compared to those receiving the supplementation from birth up to six months of age [171]. These findings could rely on the fact that the adaptive immune system is not fully mature during the first months of life [172], which might result in the lack of favorable immunomodulatory effects exerted by vitamin D. Interestingly, weekly fatty fish consumption (≥1 serving per week) has also been associated with a significant reduction in the risk of latent autoimmune diabetes in adults (LADA) [173].

Overall, these findings indicate that vitamin D deficiency may play a pivotal role as an environmental (nutritional) risk factor for islet autoimmunity and T1D. Moreover, vitamin D supplementation may have a protective role against T1D development, particularly during early childhood. On the other hand, the impact of vitamin D status and vitamin D supplementation during pregnancy on the offspring risk of T1D is still under debate. However, it is worth noting that the majority of the aforementioned studies are observational studies assessing the vitamin D intake mainly by retrospectively-collected data or interviews and/or through self-administered food frequency questionnaires. Data on vitamin D supplementation dose and duration, as well as on serum 25(OH)D levels at baseline and during the follow-up are lacking in most of these studies. Large randomized controlled trials with long-term follow-up and assessment of vitamin D status throughout the study duration are therefore warranted in order to establish the cause and effect relationship between vitamin D deficiency and T1D, along with the efficacy of vitamin D supplementation during fetal development and early childhood or adulthood in reducing the risk of T1D. 

## 8. Vitamin D Supplementation as an Immunomodulatory Therapy for T1D: Clinical Evidence

The efficacy of vitamin D in halting or reversing islet autoimmunity observed in animal models, as well as the epidemiologic evidence for a potential contribution of vitamin D deficiency to T1D pathogenesis prompted researchers to investigate the role of vitamin D supplementation as an adjuvant immunomodulatory therapy for the treatment of T1D. Different doses, formulations, and analogues of vitamin D have been investigated across studies (Table 1).

## 9. Cholecalciferol (Vitamin D3)

The proof of concept for a protective immunological effect of vitamin D in subjects with T1D has been first provided by Gabbay et al. [174], who demonstrated that 12-month cholecalciferol supplementation (2000 IU/day) led to a significant increase in the percentage of Tregs in patients with new-onset T1D. A similar effect was observed in a randomized controlled trial conducted on patients with a longer duration of T1D (median disease duration of 12.3 years; standard deviation, 2.8–24.5 years), which demonstrated that three-month cholecalciferol administration (4000 IU/day) significantly increased Tregs percentage, although only in males [175]. Cholecalciferol has also been shown to improve the suppressor function of Tregs. In fact, Treiber et al. [176] showed that 12-month cholecalciferol supplementation (70 IU/kg body weight/day) was able to significantly improve the suppressive capacity of Tregs in patients with new-onset T1D. 

Different interventional studies and randomized controlled trials suggest beneficial effects from vitamin D supplementation in T1D patients in terms of preservation of residual beta-cell function and glycemic control. Gabbay et al. [174] enrolled thirty-eight young patients with new-onset T1D (disease duration less than six months) and fasting or stimulated serum C-peptide levels ≥0.6 ng/mL. The participants were randomized to receive oral cholecalciferol (2000 IU/day) or placebo in addition to insulin therapy for 18 months. Interestingly, the authors showed a significant decrease in HbA1c levels at six months, along with a significant decrease in GAD65 antibody titers at 18 months in the cholecalciferol group compared to the placebo group. Moreover, stimulated serum C-peptide (assessed by two-hour mixed meal tolerance test, MMTT) was enhanced during the first 12 months (12% vs. −35%, *p* = 0.01) and had less decay until 18 months (−14% vs. −46%, *p* = 0.03) in the cholecalciferol group compared to the placebo group, indicating a potential role of cholecalciferol in effectively slowing the rate of decline in C-peptide levels in subjects with new-onset T1D. Thereafter, Mishra et al. [177] reported a trend, although not significant, towards a lower decline in residual beta-cell function—assessed by stimulated C-peptide levels—in children with established T1D supplemented with cholecalciferol (2000 IU/day) plus calcium (25 mg/kg/day) for six months. Additionally, one interventional study and one retrospective study conducted on T1D patients revealed that three-month cholecalciferol adjuvant therapy at different doses (400 up to 6000 IU/day) led to a significant improvement in glycemic control, resulting in lower post-treatment HbA1c levels [175,178]. However, C-peptide levels were not reported [178] or did not change significantly [175]. A recent interventional prospective study has demonstrated that adjuvant cholecalciferol supplementation at a dose of 3000 IU/day for 12 months is associated with improvement in glycemic control and slower decline of residual beta-cell function in children with T1D (baseline stimulated C-peptide levels >0.5 ng/mL, assessed by two-hour MMTT; duration of disease between one and two years) [179]. At the end of the study, children in the intervention group (cholecalciferol in addition to insulin therapy) exhibited significantly lower mean levels of fasting blood glucose, HbA1c, and total daily insulin doses, along with greater mean levels of stimulated C-peptide compared to the control group, who received insulin therapy alone (0.85 ± 1.01 vs. 0.31 ± 0.37, respectively). Importantly, the mean serum 25(OH)D levels in the intervention group remained within the sufficient range (>30 ng/mL) at all follow-up visits [179].

Conversely, Shih et al. [180] reported that cholecalciferol supplementation at a dose of 20,000 IU/week for six months did not affect HbA1c, total daily insulin doses, and serum levels of inflammatory markers in adolescents with established T1D. However, the results of this study may have been affected by some limitations, including the small sample size (n = 25), the lack of assessment of serum C-peptide levels, and the enrollment of subjects with a duration of the disease well above one year (mean disease duration at baseline in vitamin D-sufficient group and vitamin D-deficient group was 8.4 ± 4.67 years and 7 ± 3.94 years, respectively) [180]. Similarly, Sharma et al. [181] showed that cholecalciferol at a single monthly dose of 60,000 IU in addition to insulin therapy (intervention group) for six months did not lead to any significant difference in HbA1c and mean insulin requirements compared to insulin therapy alone (control group) in children with established T1D. However, the authors found a significant increase in mean fasting C-peptide levels in the intervention group compared to the control group (0.51 vs. 0.33 ng/mL, respectively; *p* < 0.05), which was also accompanied by significantly higher mean serum levels of 25(OH)D (68.64 ± 24.2 vs. 19.13 ± 7.9 ng/mL, *p* < 0.01) [181]. Furthermore, Perchard et al. [182] demonstrated that a single oral cholecalciferol dose of 100,000 or 160,000 IU did not result in any significant difference in mean HbA1c in children with vitamin D deficiency and established T1D. Nonetheless, serum levels of C-peptide were not assessed in the study, and serum 25(OH)D levels were available only for a small proportion of patients during the follow-up [182]. Additionally, it is likely that the use of a single high dose of oral cholecalciferol was not able to maintain sufficient serum 25(OH)D levels over a long follow-up period, as it has been previously demonstrated [183]. 

## 10. Cholecalciferol in Combination with Anti-Inflammatory or Anti-Hyperglycemic Agents

Some case studies conducted in patients with T1D documented potential protective effects on beta-cell function deriving from the use of cholecalciferol in addition to anti-inflammatory or anti-hyperglycemic agents, such as omega-3 polyunsaturated fatty acids (PUFA) or dipeptidyl peptidase-4 (DPP-4) inhibitors. 

Omega-3 PUFAs (especially eicosapentaenoic acid (EPA) and docosahexaenoic acid (DHA)) exert anti-inflammatory properties [184] acting as the precursors of specific lipid mediators involved in the resolution of inflammation, also known as resolvins (or SPMs, specialized pro-resolving lipid mediators) [185]. On the contrary, omega-6 PUFAs (especially arachidonic acid, AA) promote inflammation through their derived metabolite eicosanoids [186,187]. Indeed, AA:EPA ratio represents a surrogate marker of omega-6:omega-3 ratio and it has been proposed as a hallmark of systemic inflammation [187,188,189]. 

Kagohashi et al. [190] showed that a diet with a low omega-6:omega-3 ratio was able to prolong survival of NOD mice when started shortly after the onset of overt diabetes. Interestingly, a nested case-control analysis conducted by Niinistö et al. [191] within the Finnish Type 1 Diabetes Prediction and Prevention Study birth cohort—which included children carrying HLA-conferred susceptibility to T1D (n = 7782)—revealed that higher serum AA:DHA ratio at three months of age and higher serum omega-6:omega-3 ratio at six months of age were significantly associated with an increased risk of islet autoimmunity. Bi et al. [192] demonstrated that dietary intervention with EPA and DHA in NOD mice led to a reduced incidence of severe insulitis and diabetes, modulating the differentiation of CD4+ T cells and Tregs and decreasing the levels of pro-inflammatory cytokines, whereas an AA-enriched diet resulted in opposing pro-inflammatory effects. Similar results were also replicated in vitro on human peripheral blood mononuclear cells [192]. Previous studies documented that intake of cod liver oil (a dietary source containing high amounts of vitamin D and omega-3 PUFAs) during pregnancy [160] and during the first year of life [171] was associated with a reduced risk of T1D later in life, suggesting potential synergistic anti-inflammatory effects of vitamin D in combination with omega-3 PUFAs. With regard to clinical studies, Haller et al. [193] showed that autologous umbilical cord blood infusion followed by 12-month supplementation with cholecalciferol (2000 IU/day) and DHA (38 mg/kg/day) failed to preserve C-peptide secretion in children with new-onset T1D. Nevertheless, three single case studies showed that cholecalciferol in addition to high-dose omega-3 PUFAs (55–70 mg of EPA and DHA/kg body weight per day) preserved residual beta-cell function and sustained partial clinical remission in children with new-onset T1D [194,195,196]. In this regard, we are currently conducting a phase I/II clinical trial (POSEIDON, Pilot Study of Omega-3 and Vitamin D in High-Dose in Type I Diabetic Patients; ClinicalTrials.gov Identifier: NCT03406897) aimed to investigate whether one-year supplementation with cholecalciferol—alone or in combination with omega-3 PUFAs (150 mg of EPA and DHA/kg body weight)—is able to halt autoimmunity and preserve beta-cell function in pediatric and adult subjects with new-onset and established T1D. Importantly, the cholecalciferol administered dose will depend on serum 25(OH)D levels of participants at baseline and will be then progressively adjusted according to the serum levels achieved by participants during the follow-up. In particular, cholecalciferol treatment will be aimed to achieve and maintain target serum 25(OH)D levels >40 ng/mL. Similarly, omega-3 PUFA supplementation will be aimed to achieve and maintain a target AA:EPA ratio of 1.5 to 3.0 [197].

DPP-4 inhibitors are currently used as oral anti-hyperglycemic agents for treatment of T2D. DPP-4 inhibitors prevent the degradation of glucagon-like peptide-1 (GLP-1) by DPP-4 enzymes and increase endogenous levels of GLP-1, which in turn promotes insulin secretion in a glucose-dependent manner [198]. Noteworthy, pre-clinical studies conducted on NOD mice demonstrated that DPP-4 inhibitors can prevent and even reverse autoimmune diabetes, stimulating beta-cell proliferation and modulating inflammatory and autoimmune responses [199,200,201]. The efficacy of DPP-4 inhibitors as additional anti-hyperglycemic agents for the management of T1D has also been investigated, although the results remain unclear [202]. However, some case studies showed a potential efficacy of cholecalciferol in combination with the DPP-4 inhibitor sitagliptin in sustaining clinical remission in new-onset T1D [203], as well as insulin independence in LADA [204]. The rationale for such combination therapy in autoimmune diabetes may essentially consist in the synergism between the DPP-4 inhibition-mediated effects on pancreatic endocrine cells (reduction in glucagon secretion from alfa cells, increase in insulin secretion from beta cells, and potential stimulation of beta-cell proliferation) and the immunomodulatory effects exerted by both vitamin D and DPP-4 [205]. Nonetheless, future prospective studies are needed to confirm this hypothesis.

## 11. Ergocalciferol (Vitamin D2)

To the best of our knowledge, no study has evaluated the efficacy of ergocalciferol in T1D. However, a randomized, placebo-controlled trial (Vitamin D and Residual Beta-Cell Function in Type 1 Diabetes; ClinicalTrials.gov Identifier: NCT03046927) is currently investigating whether 12-month ergocalciferol supplementation is able to increase residual beta-cell function and prolong the duration of the partial clinical remission in children with new-onset T1D who are on standardized insulin treatment. 

## 12. Calcidiol (25-Hydroxyvitamin D3)

Federico et al. [206] investigated the immunomodulatory effect of one-year calcidiol supplementation in eight children with new-onset T1D (mean disease duration: 0.7 ± 0.2 years) and vitamin D deficiency. The supplementation was aimed to achieve and maintain target serum 25(OH)D levels in the range 50–80 ng/mL. By two months of supplementation, subjects showed mean circulating 25(OH)D levels in the desired range (71 ± 6 ng/mL), which remained stable at one year (71 ± 5 ng/mL). At two months, authors found a significant reduction in peripheral blood mononuclear cell reactivity against GAD-65 and proinsulin, which was inversely correlated with serum 25(OH)D concentrations. Moreover, fasting C-peptide levels remained stable after one year of treatment [206]. 

A possible advantage of calcidiol administration in T1D may rely on the fact that immune cells express 1α-hydroxylase and are therefore capable to locally convert 25(OH)D3 into 1,25(OH)2D3. Hence, calcidiol supplementation may not significantly affect circulating levels of 1,25(OH)2D3, resulting in a concomitant reduced risk of hypercalcemia compared to calcitriol administration. In agreement with this, Federico et al. [206] did not observe any significant change in circulating levels of 1,25(OH)2D. Moreover, serum calcium levels remained within the normal range at the end of the study and adverse side effects were not reported.

## 13. Calcitriol (1,25-Dihydroxyvitamin D3) 

The studies using calcitriol in addition to insulin therapy for treatment of T1D have been disappointing. Pitocco et al. [207] first conducted an open-label randomized trial investigating the effect of calcitriol (at a dose of 0.25 μg on alternate days) or nicotinamide (25 mg/kg daily) on residual beta-cell function and glycemic control in patients with new-onset T1D followed up for one year. Although calcitriol administration was temporarily associated with a significant reduction in insulin requirement (up to six months), the authors did not find significant changes in fasting and stimulated C-peptide and HbA1c levels at the end of the study. Thereafter, two different randomized placebo-controlled trials demonstrated that calcitriol supplementation at a dose of 0.25 μg/day for 18 to 24 months is safe but ineffective in preserving residual beta-cell function and improving glycemic control in patients with new-onset T1D and baseline fasting C-peptide levels >0.25 nmol/L (>0.75 ng/mL) [208,209]. 

The negative findings may be attributed to the relatively low doses of calcitriol administered in the aforementioned studies in order to avoid the risk of adverse side effects related to hypercalcemia. Moreover, the short serum half-life of calcitriol might result in fluctuations in its serum concentration and subsequent lack of immunomodulatory effects on beta cells. This could therefore explain the better results obtained from studies on the use of cholecalciferol in T1D, which did not report the development of hypercalcemia and serious adverse side effects even under high dose supplementation.

## 14. Alfacalcidol (1α-hydroxycholecalciferol)

A few studies have investigated the efficacy of the vitamin D analogue alfacalcidol in addition to insulin therapy for treatment of T1D. Alfacalcidol (1α-hydroxycholecalciferol) is an analogue of vitamin D, which acts as a prodrug of the active form calcitriol. In order to be converted into calcitriol, alfacalcidol is activated by the vitamin D-25-hydroxylase enzyme in the liver, without need for the second hydroxylation step in the kidney. It has long been used for the treatment of hypocalcemia, end-stage renal disease, hypoparathyroidism, and osteoporosis [210]. However, alfacalcidol has also been shown to exert anti-inflammatory and immunomodulatory effects [211,212]. One trial conducted on newly-diagnosed T1D children suggested that alfacalcidol (at a dose of 0.25 μg twice daily) can preserve beta-cell function, as evidenced by higher fasting C-peptide levels and lower daily insulin doses observed in the intervention group [213]. Similarly, Li et al. [214] documented that one-year treatment with alfacalcidol (at a dose of 0.5 μg/day) in addition to insulin therapy is associated with a partial preservation of beta-cell function in patients with LADA. Notably, patients with a shorter duration of disease (less than one year) exhibited a better response to alfacalcidol in terms of preservation of fasting- and post-prandial C-peptide levels in comparison to patients treated with insulin therapy alone [214]. However, the small sample size of these studies does not allow definitive conclusions to be made on the efficacy of alfacalcidol in autoimmune diabetes. Hence, future prospective interventional studies are warranted. 

## 15. Discussion

Over the last decades, it has become clear that vitamin D action goes well beyond the regulation of calcium homeostasis and bone metabolism. In this regard, the anti-inflammatory and immunomodulatory effects of vitamin D have been clearly demonstrated in animals and humans. A growing body of evidence also indicates that vitamin D deficiency could play a role in the pathogenesis of T1D [82], suggesting hypovitaminosis D as an important environmental factor for development of the disease. Indeed, several studies have documented that vitamin D intake—especially during early childhood—can reduce the risk of developing T1D later in life. Therefore, vitamin D deficiency should be promptly diagnosed and properly treated during the first years of life, especially in children at high genetic risk for T1D (as defined by family history of T1D and islet autoantibody and/or HLA DR3/DR4 positivity). Furthermore, it has been widely shown that vitamin D deficiency is more prevalent and more profound in patients with newly-diagnosed and long-standing T1D compared to healthy controls.

Since the prevalence of vitamin D deficiency is dramatically increasing at a global level [39], nutritional strategies to increase vitamin D dietary intake and counteract this epidemiologic trend are deemed necessary, especially in regions where sun exposure is limited by latitude or lifestyle-related factors. The UK Scientific Advisory Committee on Nutrition (SACN) recommends a dietary intake of vitamin D of 10 μg/day (400 IU/day) for the general population aged four years and older [215]. The Endocrine Society Guidelines on prevention of vitamin D deficiency recommend a minimum dietary intake of vitamin D of 400 IU/day for infants (up to 1 year of age), and 600 IU/day for children, adolescents, and adults [216].

However, maintaining the recommended daily intakes only from natural food sources is challenging due to the paucity of foods containing high amounts of vitamin D (e.g., fatty fish, cod liver oil, egg yolks) [39,48]. On the other hand, the low adherence to supplementation may affect the long-term efficacy of vitamin D supplements in prevention and treatment of vitamin D deficiency. In this context, food fortification has been suggested as a safe and cost-effective strategy to consume the recommended daily amount of vitamin D [217,218]. In general, food can be enriched with vitamin D through two different approaches: (i) traditional fortification, which consists of direct addition of vitamin D into food, and (ii) biofortification (or bio-addition), which refers to various strategies to indirectly increase vitamin D content of food (e.g., fortifying animal diets with vitamin D or UV light exposure of yeast and mushrooms) [218,219]. Therefore, further investigation is warranted to assess whether vitamin D-fortified foods (e.g., bread, milk, dairy products, flour, cereals, and cooking oils, which are highly consumed worldwide) represent an effective tool to increase vitamin D daily intake and long-term treatment of vitamin D deficiency.

The rationale for investigation of vitamin D as an adjuvant immunomodulatory therapy in T1D essentially relies on its potential ability to restore immune tolerance, counteract autoimmune response, slow down or halt disease progression, preserve residual beta-cell mass and function, and improve glycemic control. Several vitamin D formulations (cholecalciferol, calcidiol, calcitriol, or alfacalcidol) have been tested in T1D to preserve beta-cell function.

Pre-clinical studies have demonstrated the efficacy of vitamin D in preventing and reversing autoimmunity in NOD mice. On the other side, clinical studies on vitamin D efficacy as an immunomodulatory agent in T1D led to inconclusive results. The possible cause of the discrepancies in the results may be related, at least in part, to heterogeneity of the study duration, along with the different vitamin D formulations and doses used across studies. However, the most promising results in terms of preservation of residual beta-cell function come from the use of cholecalciferol and alfacalcidol [220], although caution is needed in making definitive conclusions due to the small sample size of the studies.

Moreover, the majority of studies assessed fasting C-peptide levels as a surrogate marker of beta-cell function. Fasting C-peptide measures basal insulin secretion and does not detect early beta-cell loss as effectively as stimulated C-peptide, which greatly limits the interpretation of several of these studies. In addition, several studies assessed clinical outcomes solely in relation to the administered dose of vitamin D, without reporting serum 25(OH)D levels at baseline and/or during follow-up. Notably, the individual serum response to a given dose of vitamin D is highly variable and depends on several factors, such as baseline vitamin D status, body fat percentage, gender, ethnicity, genetics, seasonal variations, medications, and type of vitamin D formulation [221]. This aspect becomes particularly crucial especially in the context of autoimmune diseases, where the immunomodulatory effects of vitamin D may be observed only upon the achievement of serum 25(OH)D levels above those required for bone health (≥30 ng/mL). In this regard, an optimal study design is critical to effectively assess the impact of vitamin D on study outcomes [222,223].

Finally, gene polymorphisms in vitamin D hydroxylases, VDBP, and VDR need to be more extensively investigated to assess their impact on vitamin D status and its mechanistic effects. This may allow for the identification of specific subgroups of subjects that may require higher vitamin D doses to achieve the desired target serum 25(OH)D levels for prevention or treatment of T1D.

## 16. Conclusions

Vitamin D deficiency may play a role in determining the risk of developing T1D in the first years of life, particularly in children at high genetic risk. Moreover, vitamin D deficiency is highly prevalent in patients with T1D. However, data on vitamin D supplementation and preservation of beta-cell function in T1D remain inconclusive. Future large-scale prospective trials are warranted to adequately assess the role of vitamin D as a disease-modifying agent in T1D.

## Figures and Tables

**Table 1:** Summary of the main studies on the use of vitamin D in addition to insulin therapy in patients with new-onset and established T1D. Abbreviations: 25(OH)D, 25-hydroxivitamin D; CRP, C-reactive protein; DHA, docosahexaenoic acid; GAD65, glutamic acid decarboxylase; HbA1c, glycated hemoglobin; IL-6, interleukin 6; LADA, latent autoimmune diabetes in adults; MCP-1, monocyte chemoattractant protein 1; n/a, not available; T1D, type 1 diabetes; TNF-α, tumor necrosis factor-alpha; Tregs, regulatory T cells; UCB, umbilical cord blood.

Study Design	Study Population	Study Treatment and Duration	Main Findings	References
Randomized, double-blind, placebo-controlled, prospective trial	*n* = 38 Mean age (intervention group and placebo group): 13.5 ± 5.1 vs. 12.5 ± 4.8 years Mean T1D duration: (intervention group and placebo group): 2.2 ± 1.2 vs. 2.7 ± 1.7 months	Patients were randomly assigned to receive cholecalciferol (2000 IU/day) or placebo for 18 months	Significant increase in Tregs percentage and MCP-1 levels at 12 months in cholecalciferol group vs. placebo group Significant decrease in HbA1c levels at six months in cholecalciferol group vs. placebo group Significant decrease in GAD65 autoantibody titers at 18 months in cholecalciferol group vs. placebo group Stimulated C-peptide was significantly enhanced during the first 12 months in cholecalciferol group vs. placebo group Subjects in the cholecalciferol group were significantly less likely to progress towards undetectable fasting C-peptide at 18 months compared to subjects in the placebo group	Gabbay et al. [174]
Randomized, double-blind, placebo-controlled trial	*n* = 30 Median age: 12 years (interquartile range, 11–16 years) Mean T1D duration (intervention group and placebo group): 61 ± 20 days vs. 61 ± 28 days	Patients were randomly assigned to receive cholecalciferol (70 IU/kg body weight/day) or placebo for 12 months	Significant improvement in suppressive capacity of Tregs in cholecalciferol group vs. placebo group	Treiber et al. [176]
Randomized, double-blind, placebo-controlled, crossover trial	*n* = 39 Median age: 44 years (interquartile range, 34–52 years) Mean T1D duration: 12.3 years (interquartile range, 2.8–24.5 years)	Patients were randomly assigned to receive either cholecalciferol (4000 IU/day) for three months and placebo for the following three months, or the sequential alternative Effects of cholecalciferol treatment were assessed based on intra-individual changes between intervention and placebo periods for outcome measures (primary outcome was a change of Tregs percentage, whereas secondary outcomes were changes in HbA1c and daily insulin requirements)	Cholecalciferol treatment was associated with a significant increase in Tregs percentage (only in males), along with a significant reduction in daily insulin requirements and HbA1c	Bogdanou et al. [175]
Prospective, case-control interventionaltrial	*n* = 30 Mean age (intervention group and control group): 10.8 ± 1.78 years vs. 9.73 ± 1.38 years Mean T1D duration: 1.12 ± 1.73 years	Fifteen T1D patients were assigned to the intervention group (cholecalciferol 2000 IU/day plus calcium 25 mg/kg/day) for six months, whereas fifteen age-matched T1D patients were enrolled and followed up as controls for six months	Patients in the intervention group showed a non-significant trend towards a lower decline in stimulated C-peptide levels at six months compared to patients in the control group	Mishra et al. [177]
Retrospective study	*n* = 73 children included in the final analysis Mean age: 7.7 ± 4.4 years Duration of T1D: n/a	Patients with serum 25(OH)D levels < 12 ng/mL * were treated with cholecalciferol 6000 IU/day for three months Patients with serum 25(OH)D levels between 12 and 20 ng/mL * were treated with cholecalciferol 400 IU/day for three months	Cholecalciferol treatment was associated with a significant reduction in HbA1c levels	Giri et al. [178]
Prospective, case-control interventionalstudy	*n* = 72 Age (inclusion criteria): six to 12 years Duration of T1D (inclusion criteria): between one and two years	Forty-two participants received cholecalciferol(3000 IU/day) in combination with insulin therapy for one year, whereas thirty age-matched controls received insulin therapy alone	Patients in cholecalciferol group exhibited significantly lower mean levels of fasting blood glucose, HbA1c and total daily insulin doses, along with greater mean levels of stimulated C-peptide compared to the control group	Panjiyar et al. [179]
Randomized, prospective, crossover study	*n* = 25 Mean age (vitamin D-sufficient group and vitamin D-deficient group): 17.2 ± 1.9 years vs. 16.2 ± 1.8 years Mean T1D duration (vitamin D-sufficient group and vitamin D-deficient group): 8.4 ± 4.67 years vs. 7.0 ± 3.94 years	Subjects received cholecalciferol (20,000 IU/week) for six months, either immediately or after six months of observation	Cholecalciferol treatment did not affect HbA1c, total daily insulin doses, and serum levels of inflammatory markers (CRP, IL-6 and TNF-α)	Shih et al. [180]
Randomized, double-blind controlled trial	*n* = 52 Mean age (intervention group and control group): 9.5 ± 3.9 vs. 9.0 ± 4.4 years Duration of T1D (intervention group and control group): 4.75 ± 3.0 vs. 4.0 ± 2.5 years	Oral cholecalciferol was administered at a dose of 60,000 IU/monthly for six months in addition to insulin therapy in the intervention group, whereas only insulin therapy was administered in the control group	Significant increase in mean fasting C-peptide levels in the intervention group compared to the control group No significant changes were observed between intervention and control group in HbA1c levels and mean daily insulin requirements	Sharma et al. [181]
Pilot interventional study	*n* = 42 Mean age: 12.5 ± 3.5 years Mean T1D duration: 4.8 ± 3.3 years	Participants with serum 25(OH)D levels < 20 ng/mL * were treated with a single oral cholecalciferol dose of 100,000 IU (two to 10 years) or 160,000 IU (>10 years)	No significant differences in mean HbA1c levels for one year before and one year after cholecalciferol treatment, or for three months before and after cholecalciferol treatment	Perchard et al. [182]
Open-label, randomized trial	*n* = 15 Median age (intervention group and control group): 7.2 vs. 6.6 years Duration of T1D: median time from diagnosis to screening was 119 days in the intervention group and 106 days in the control group	Participants received either autologous UCB infusion followed by 12-month supplementation with oral cholecalciferol (2000 IU/day) and DHA (38 mg/kg/day) plus intensive diabetes management (intervention group), or intensive diabetes management alone (control group)	Area under the curve C-peptide declined and daily insulin doses increased in both groups compared to baseline No significant differences were observed between groups in terms of HbA1c levels, Tregs frequency, total CD4 counts, and autoantibody titers	Haller et al. [193]
Pilot interventional study	*n* = 15 patients Mean age: 12 ± 0.9 years Mean T1D duration: 0.7 ± 0.2 years	Eight patients with vitamin D deficiency (out of fifteen consecutive T1D patients) received calcidiol to achieve and maintain serum 25(OH)D levels between 50 and 80 ng/mL for up to one year. The remaining seven patients with serum 25(OH)D levels ≥20 ng/mL were not supplemented Starting calcidiol dose was 10 μg/day. Calcidiol dose was progressively adjusted (up to 28 ± 8.2 μg/day) until serum 25(OH)D levels were steadily in the desired range (50–80 ng/mL)	Significant reduction in peripheral blood mononuclear cell reactivity against GAD65 and proinsulin was observed in the supplemented group at two months Fasting C-peptide levels remained stable after one-year treatment with calcidiol	Federico et al. [206]
Open-label, randomized controlled trial	*n* = 70 Mean age: 13.6 ± 7.6 years Duration of T1D (inclusion criteria): <four weeks	Participants were randomized to receive calcitriol (0.25 μg on alternate days) or nicotinamide (25 mg/kg/day) and followed up for one year	Calcitriol treatment was temporarily associated with a significant reduction in daily insulin requirements (up to six months) No significant differences were observed between calcitriol and nicotinamide groups in terms of fasting and stimulated C-peptide and HbA1c levels	Pitocco et al. [207]
Randomized, double-blind, placebo-controlled trial	*n* = 40 Median age: 31.4 ± 6.8 years Median T1D duration (intervention group and placebo group): 35 days vs. 40 days	Participants were randomly assigned to calcitriol (0.25 μg/day) or placebo for nine months and followed up for a total of 18 months	No significant differences were observed between groups in terms of fasting and stimulated C-peptide levels and daily insulin requirements	Walter et al. [208]
Randomized, double-blind, placebo-controlled trial	*n* = 27 Median age: 18 years Duration of T1D (inclusion criteria): <12 weeks	Participants were randomized to receive calcitriol (0.25 μg/day) or placebo and followed up for two years	No significant differences were observed between groups in terms of fasting and stimulated C-peptide levels, HbA1c levels and daily insulin requirements	Bizzarri et al. [209]
Randomized, single-blind, placebo-controlled trial	*n* = 54 Mean age: 10.1 ± 2.1 years Mean T1D duration: 43 ± 15 days	Participants were randomized to receive alfacalcidol (0.25 μg twice daily) or placebo for six months	Participants in alfacalcidol group showed significantly higher fasting C-peptide levels and lower daily insulin requirements compared to placebo group	Ataie-Jafari et al. [213]
Prospective randomized controlled trial	*n* = 35 (LADA patients) Mean age (insulin group and insulin plus alfacalcidol group): 42.8 ± 12.9 years vs. 38.5 ± 12.5 years Median duration of LADA (insulin group and insulin plus alfacalcidol group): 0.5 years vs. one year	Participants were randomly assigned to receive insulin therapy alone or insulin therapy plus alfacalcidol (0.5 μg/day) for one year	70% of patients treated with alfacalcidol maintained or increased fasting C-peptide levels after one year of treatment, whereas only 22% of patients treated with insulin therapy alone maintained stable fasting C-peptide levels Subgroup analysis showed that patients with a shorter disease duration (<one year) in the alfacalcidol plus insulin group exhibited significantly higher fasting and post-prandial C-peptide levels	Li et al. [214]

* Values converted from nmol/L to ng/mL.
